# Treatment patterns of antidepressants in children and adolescents in Scandinavia

**DOI:** 10.1007/s00787-024-02433-7

**Published:** 2024-04-29

**Authors:** Lotte Rasmussen, Peter Bjødstrup Jensen, Johan Reutfors, Kari Furu, Svetlana Skurtveit, Randi Selmer, Per Damkier, Mette Bliddal, Rikke Wesselhoeft

**Affiliations:** 1 Clinical Pharmacology, Pharmacy and Environmental Medicine, Department of Public Health, University of Southern Denmark, JB Winsløws Vej 19, 5000 Odense, Denmark; 2 Research Unit of Child and Adolescent Mental Health, Institute for Clinical Research, University of Southern Denmark, Odense, Denmark; 3 Centre for Pharmacoepidemiology, Clinical Epidemiology Division, Department of Medicine Solna, Karolinska Institutet, Stockholm, Sweden; 4 Department of Chronic Diseases, Norwegian Institute of Public Health, Oslo, Norway; 5 Department of Clinical Pharmacology, Odense University Hospital, 5000 Odense C, Denmark; 6 Department of Clinical Research, University of Southern Denmark, 5000 Odense, Denmark; 7 OPEN Research Unit, Department of Clinical Research, University of Southern Denmark, 5000 Odense C, Denmark

**Keywords:** Antidepressants, Drug utilization, Denmark, Norway, Sweden, Children and adolescents

## Abstract

The aim of this study was to examine variations in use of antidepressants among children and adolescents in the three Scandinavian countries (Sweden, Norway, and Denmark). We identified new users of antidepressants (5–17 years) during 2007–2018 and described the annual incidence rate, treatment duration, concomitant psychotropic drug use, and the clinical setting of the prescribing physician (in Sweden and Denmark). Incident use of antidepressants increased by a factor 1.9 in Sweden, 1.3 in Norway and decreased by a factor 0.6 in Denmark during the study period. In Sweden, 58% of antidepressant users were covered by a prescription 12 months after initiation compared to 40% in Norway and 49% in Denmark. Also, 34% of Swedish antidepressant users were in continuous treatment after 12 months compared to 26% in Norway and 31% in Denmark. Concomitant use of other psychotropics was more common in Sweden (57%) than in Norway (37%) and Denmark (27%). During 2007–2018, clinicians from psychiatry settings initiated 75% of antidepressant treatments in Sweden, while this was the case for 50% of prescriptions in Denmark, although the proportion increased over time. The number of new antidepressant users is high and still rising in Sweden compared to Norway and Denmark. Swedish antidepressant users are more likely to use other psychotropics and to be covered by an antidepressant prescription after one year. Most antidepressants in Sweden are prescribed by physicians within psychiatric settings suggesting that they are based on specialized psychiatric evaluation.

**Supplementary Information:**

The online version contains supplementary material available at 10.1007/s00787-024-02433-7.

## Introduction

Use of antidepressants in children and adolescents is debated due to the association between treatment with selective serotonin reuptake inhibitors (SSRIs) and increased risk of suicidal ideation and behavior [1–5].

Real-world data on the use of antidepressants are essential to inform health regulators and guide future initiatives to facilitate rational pharmacotherapy. Most epidemiological studies providing real-world evidence on antidepressant use have included data up until 2012 [6], showing increasing or u-shaped trends. From 2005 to 2012, the prevalence of antidepressant use increased by 25% in the United States, 54% in the United Kingdom, 18% in the Netherlands, and 60% in Denmark among children and adolescents [7]. Similarly, the prevalence of antidepressant use increased by 30% from 2004 to 2013 in Norway, particularly among girls aged 16–17 years [8].

We have previously documented large variations in the prevalence of antidepressant use in children and adolescents across the Scandinavian countries [9]. Specifically, we found that the prevalence of antidepressant use decreased in Denmark from 2010 onwards, while it increased slightly in Norway and doubled in Sweden during the same period [9]. The reasons for these variations are unknown and warrant further investigation especially when considering the similar universal health care systems [10] and identical licensing status of antidepressants in Scandinavia [11].

The existing literature has examined antidepressant use among youths from different countries at an overall level. Detailed information on the specific treatment patterns and characteristics of users as well as clinical practice across countries will take us a step further in understanding the reasons for cross-national variations in antidepressant drug use.

The aim of this study was to examine variations in patterns of antidepressant drug use among children and adolescents in Sweden, Norway, and Denmark. Based on the Scandinavian health registers, we focused on the following measures: incidence rates of use, use of specific antidepressants, duration of antidepressant treatment, concomitant psychotropic drug use, and the clinical setting of the prescribing physician.

## Methods

### Data sources

We identified our study population based on individual-level data from national prescription registries in Sweden [12], Norway [13], and Denmark [14]. The Scandinavian national prescription registries hold information on all drugs dispensed at pharmacies to outpatients [15]. Information used in our study included the drug dispensing date, the Anatomical Therapeutic Chemical (ATC) classification code, the volume of dispensed drug expressed in Defined Daily Doses (DDD), package size, and the clinical setting of the prescribing physician (only available in Sweden and Denmark). The ATC and the DDD systems were developed and are maintained by the World Health Organization (WHO) Collaborating Centre for Drug Statistics Methodology [16]. We obtained information on total population counts from Statistics Sweden, Statistics Norway, and Statistics Denmark.

### Study population and study drugs

Our study population included all children and adolescents who were new users of antidepressants between January 1st, 2007, and December 31st, 2018, in Sweden, Norway, and Denmark. Use was defined as filling of at least one prescription. Individuals were considered new users if they had not filled an antidepressant prescription since 2005 in all countries allowing for identical wash-out periods across countries. We only included children and adolescents who were aged 5–17 years at treatment initiation. Children under the age of 5 were excluded due to low use of antidepressants [7]. Antidepressants included all drugs coded under the ATC-code N06A and they were further divided into SSRIs (ATC N06AB), tricyclic antidepressants (TCAs) (N06AA), and other antidepressants (N06AX, N06AF, and N06AG).

### Analysis

We performed six analyses that were all stratified by country. First, we described the population of new antidepressant users according to age at treatment initiation, sex, and the number of tablets (package size) dispensed at the first filled prescription. If multiple packages were dispensed on the same day, the largest package size was reported.

Second, we estimated the annual incidence rate of antidepressant use from January 1st, 2007, to December 31st, 2018. The incidence rate was calculated by dividing the number of new users of antidepressants by the total follow-up time in the population in the given year. Due to a negligible number of new users compared to the total population count, we did not remove individuals that were no longer at risk from the denominator. This analysis was stratified by antidepressant classes: all antidepressants, SSRIs, TCAs, and other antidepressants, and by age and sex. A person could only enter the overall analysis once as a new user but could be a new user of more than one antidepressant drug class, e.g., be a new user of SSRIs and TCAs. We reported the relative change in the incidence rate of antidepressant use across calendar years in each country by calculating the ratio between the incidence rate in the first and last study year. To quantify the difference between boys and girls, we calculated a female/male ratio of the incidence rate.

Third, we identified the most common antidepressants prescribed to new users from January 1st, 2007, to December 31st, 2018, and reported the relative distribution among all new antidepressant prescriptions. An individual could only enter the analysis with one prescription and the analysis therefore reflects the first initiated treatment.

Fourth, we estimated antidepressant treatment duration by (1) calculating the proportion of children covered by an antidepressant prescription after 6 months and 12 months and (2) the proportion of children in continuous treatment after 6 months and 12 months, i.e., individuals with no treatment breaks. We used the ‘proportion of patients covered’ (PPC) method, described in detail elsewhere [17], to estimate the proportion of children covered by treatment after 6 months and 12 months. We used a Kaplan–Meier survival analysis to estimate the proportion of children in continuous treatment after 6 months and 12 months, i.e., with no gaps between prescriptions that exceeded the estimated duration of the single prescription. In all analyses, the single prescription duration assumed the consumption of one tablet per day while adding a grace period of 25% to account for non-compliance and stockpiling. If no prescription was refilled within the duration of the single prescription and the added grace period, the treatment was considered discontinued. We only considered individuals who initiated treatment with oral solid formulations of antidepressants and disregarded oral suspensions that constituted a negligible proportion of the total number of antidepressant prescriptions (≤ 2%). Individuals were censored at death, migration, or end of study period. This analysis was stratified by age and sex.

Fifth, to investigate concomitant use of other psychotropic drugs, we calculated the proportion of antidepressant users who filled at least one prescription for another psychotropic drug during the three months before or after initiation of antidepressant treatment, stratified by age and sex. The psychotropic drug groups considered were psychostimulants (ATC N06B), antipsychotics (N05A), anxiolytics (N05B), and hypnotics (N05C excl. N05CH01 (melatonin) and melatonin (N05CH01). In this analysis, we excluded individuals with less than 3 months follow-up after initiation of antidepressant treatment.

Finally, we identified the clinical setting of the prescriber of the first antidepressant prescription in Sweden and Denmark. The clinical setting of prescribers was divided into four categories: general practice, psychiatry (adult and child/adolescent psychiatry), pediatrics, and others (including, but not restricted to neurology). Prescriptions with missing or invalid information on the clinical setting of the prescriber were reported. For this analysis, we excluded individuals initiating at least two prescriptions at the same date (< 1%). To investigate changes over time in the distribution of the clinical setting of prescribers, we repeated the analysis restricting to patients initiating treatment in 2007–2012 and 2013–2018 respectively.

### Statistical analysis

The statistical analysis was based on a common data model where data from each country was transformed into a common data structure. Data from each country was analyzed separately following a common programming script. Finally, aggregated results were pooled for a combined analysis by the coordinating study site (Denmark). All statistical analyses were performed using STATA Release 17.0 (StataCorp, College Station, TX, USA) and R (R Core Team, 2018), including the tidyverse packages (Wickham, 2017).

### Ethical approval

The study was approved by the Danish Data Protection Agency (10.080). According to Danish legislation, approval from an ethics Committee is not required for registry-based studies. The Regional Committee for Medical Research Ethics (2010/131) and the Norwegian Data Protection Authority (10/00447-5) approved the register linkage. The use of Swedish data for this study was approved by the Swedish Ethical Review Authority (no. 2017/1236–31/2).

## Results

### Study population characteristics

Across Scandinavia, a total of 119,747 children and adolescents aged 5–17 years filled a first prescription of an antidepressant between January 1st, 2007, and December 31st, 2018. Swedish children and adolescents were slightly younger when initiating antidepressants (median 15 years) compared to Danish and Norwegian peers (median of 16 years) (Table 1).

**Table 1:** Study and patient characteristics for new users of antidepressants in Sweden, Norway, and Denmark from 2007–2018

	Total	Sweden	Norway	Denmark
*Total number of incident antidepressant users 2007—2018 (n)*	119,747	70,573	23,978	25,196
Male	42,854 (36%)	26,294 (37%)	8,096 (34%)	8464 (34%)
Female	76,893 (64%)	44,279 (63%)	15,882 (66%)	16,732 (66%)
*Age at first prescription (median, IQR)*		15 (13–16)	16 (15–17)	16 (14–17)
5–9 years (n, %)	4150 (3%)	2927 (4%)	584 (2%)	639 (3%)
10–13 years (n, %)	21,515 (18%)	15,256 (22%)	2659 (11%)	3600 (14%)
14–17 years (n, %)	94,082 (79%)	52,390 (74%)	20,735 (86%)	20,957 (83%)
*Package size at first prescription*				
≤ 30 tablets (n, %)	67,833 (57%)	45,095 (63.9%)	7,505 (31.3%)	15,233 (60.5%)
31–100 tablets (n, %)	50,663 (42%)	24,926 (35.3%)	15,942 (66.5%)	9795 (38.9%)
101–182 tablets (n, %)	571 (0%)	330 (0.5%)	201 (0.8%)	40 (0.2%)
≥ 183 tablets (n, %)	680 (1%)	222 (0.3%)	330 (1.4%)	128 (0.5%)

### Incidence rate

**Fig. 1:**
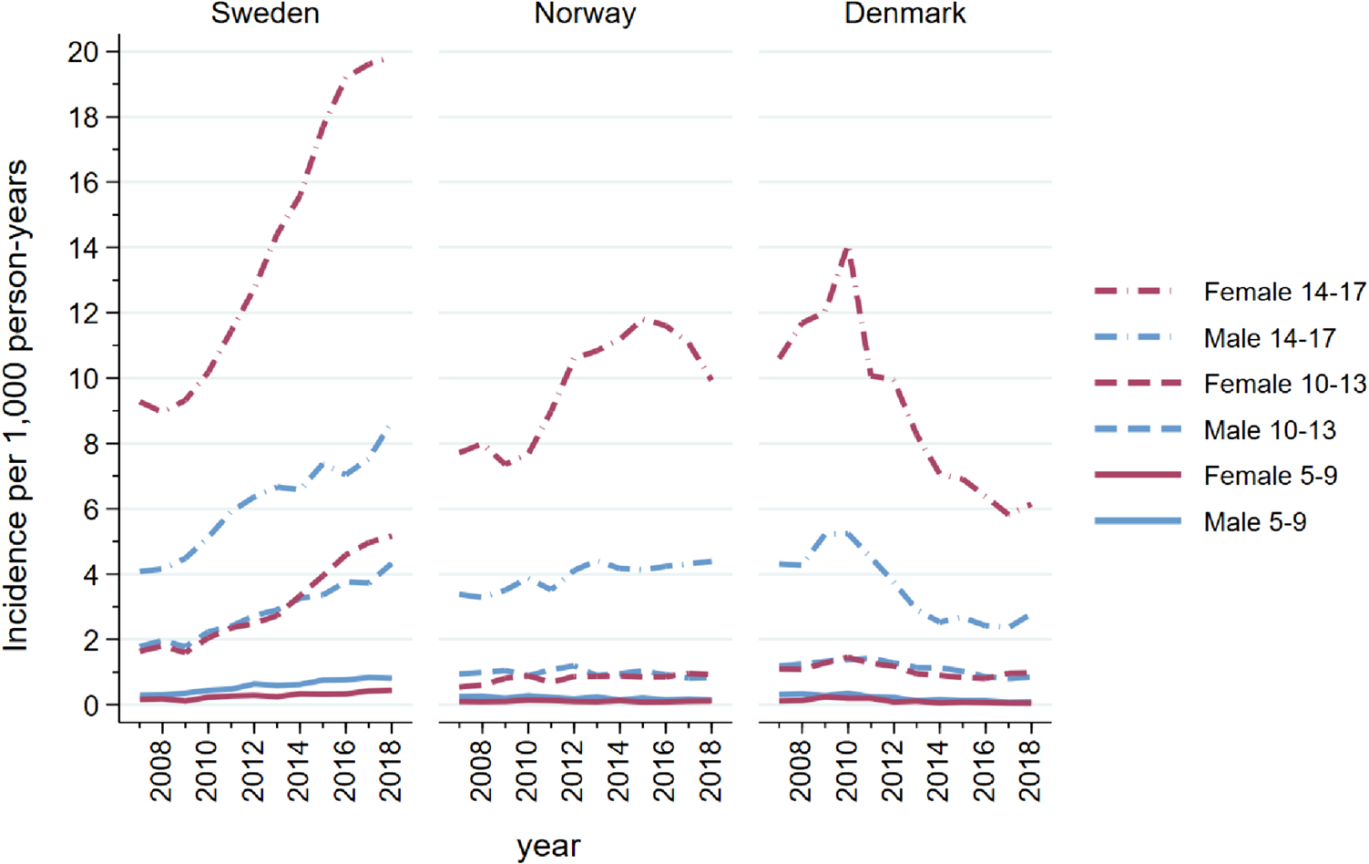
Incidence rate (new users per 1000 person-years) of antidepressant use in Sweden, Norway, and Denmark from 2007 to 2018 stratified by age and sex

From January 1st, 2007, to December 31st, 2018, the incidence rate of antidepressant use increased by a factor 1.9 in Sweden [from 3.0 to 5.8 per 1000 person-years (py)], 1.3 in Norway from 2.0 to 2.5 per 1000 py) and decreased by a factor 0.6 in Denmark (from 2.7 to 1.7 per 1000 py) (Figure S1a and Table S1a–c). Swedish children and adolescents were at least twice as likely to initiate antidepressants (5.8 per 1000 py) in 2018 compared to Danish (1.7 per 1000 py) and Norwegian (2.5 per 1000 py) peers. The incidence was highest among girls aged 14–17 years in all countries (Fig. 1). In 2018, Swedish 14–17-year-old girls had the highest incidence (20 per 1000 py), Danish girls had the lowest (6.2 per 1000 py) while Norwegian girls were in-between (9.9 per 1000 py) after a peak in incidence around 2015. The female/male ratio for adolescents aged 14–17 years decreased slightly during the study period from 2.5 to 2.2 in Denmark but was stable at 2.3 in both Norway and Sweden. The incidence among children aged 10–13 years was low and stable throughout the study period in Denmark and Norway, while it increased by a factor 2.8 for Swedish children during the study period (from 1.7 to 4.7 per 1000) (Fig. 1 and Table S1a–c). Across all countries, the rise in incident use of antidepressants was mainly driven by SSRIs (Fig. S1).

### Commonly used antidepressants

**Fig. 2:**
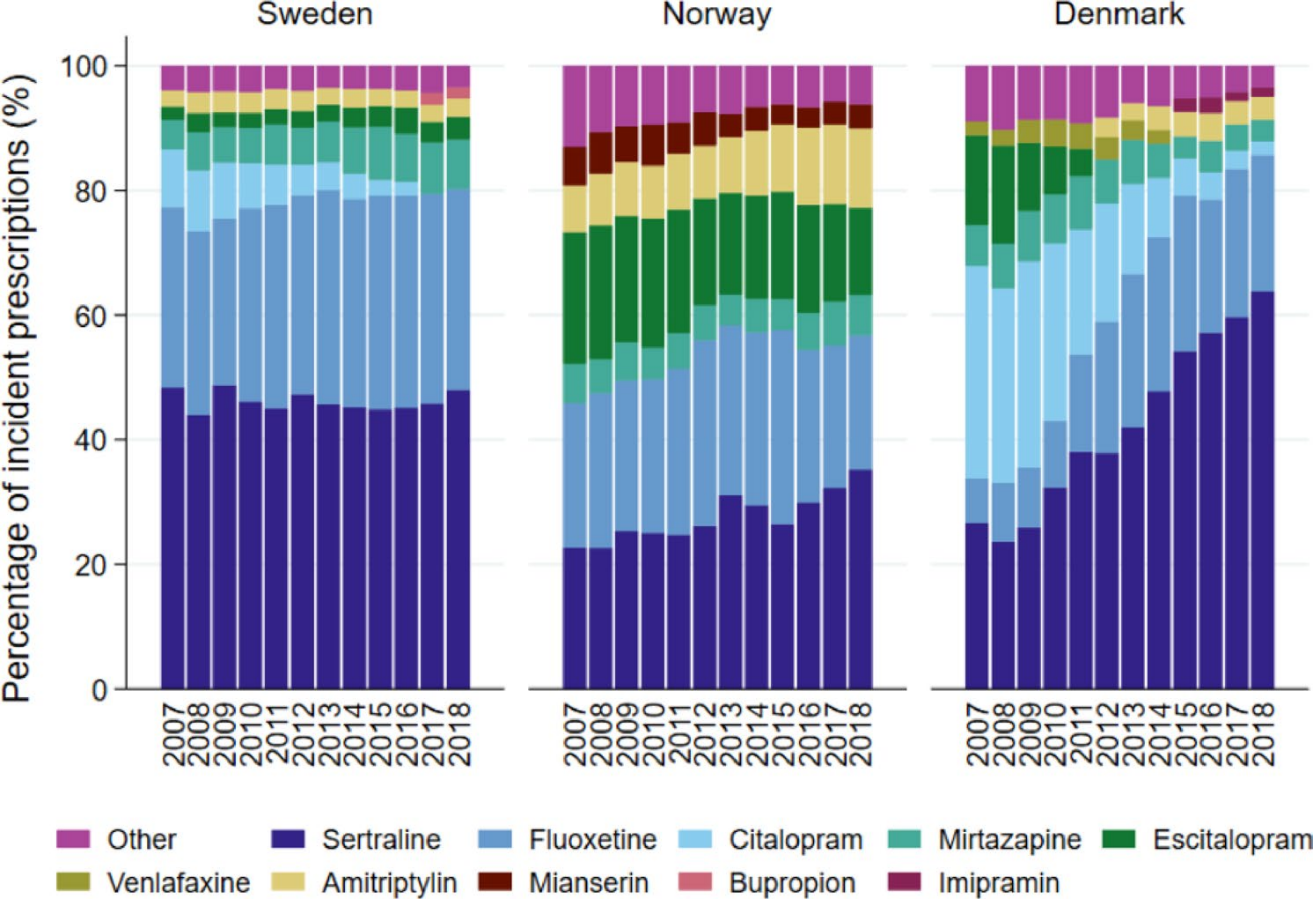
Distribution (%) of antidepressants* prescribed at first prescription from 2007 to 2018 in Sweden, Norway, and Denmark. *Each column/study year contains a maximum of six different antidepressants and an “other” category. The antidepressants included in “other” can therefore vary between study years and countries and include all antidepressants not shown in the specific columns

Sertraline was the most common antidepressant prescribed to new users, representing almost half of all new prescriptions in 2018 in Sweden (48%), two thirds in Denmark (64%), and one out of three prescriptions in Norway (35%) (Fig. 2). While sertraline and fluoxetine accounted for more than 80% of incident prescriptions in 2018 in Sweden and Denmark, the use of antidepressants among incident users was mixed in Norway with a higher use of especially escitalopram (14%), amitriptyline (13%) and mianserine (4%) compared to Denmark and Sweden. Changes over time in the proportional distribution of specific antidepressants were most notable in Denmark while there were only small changes over time in Sweden and Norway (Fig. 2).

### Treatment duration

If assuming the use of one antidepressant tablet per day, the proportion of youths covered by antidepressant treatment (measured by the PPC method) 12 months after treatment initiation was higher in Sweden (58%) compared to Norway (40%) and Denmark (49%) (Table S2). Similarly, the proportion of children and adolescents in continued antidepressant treatment (measured by the Kaplan–Meier survival analysis) after 12 months was slightly higher in Sweden (34%), than in Norway (26%) and Denmark (31%) (Table S2). Treatment duration was similar between boys and girls (data not shown).

### Concomitant use of psychotropic drugs

**Fig. 3:**
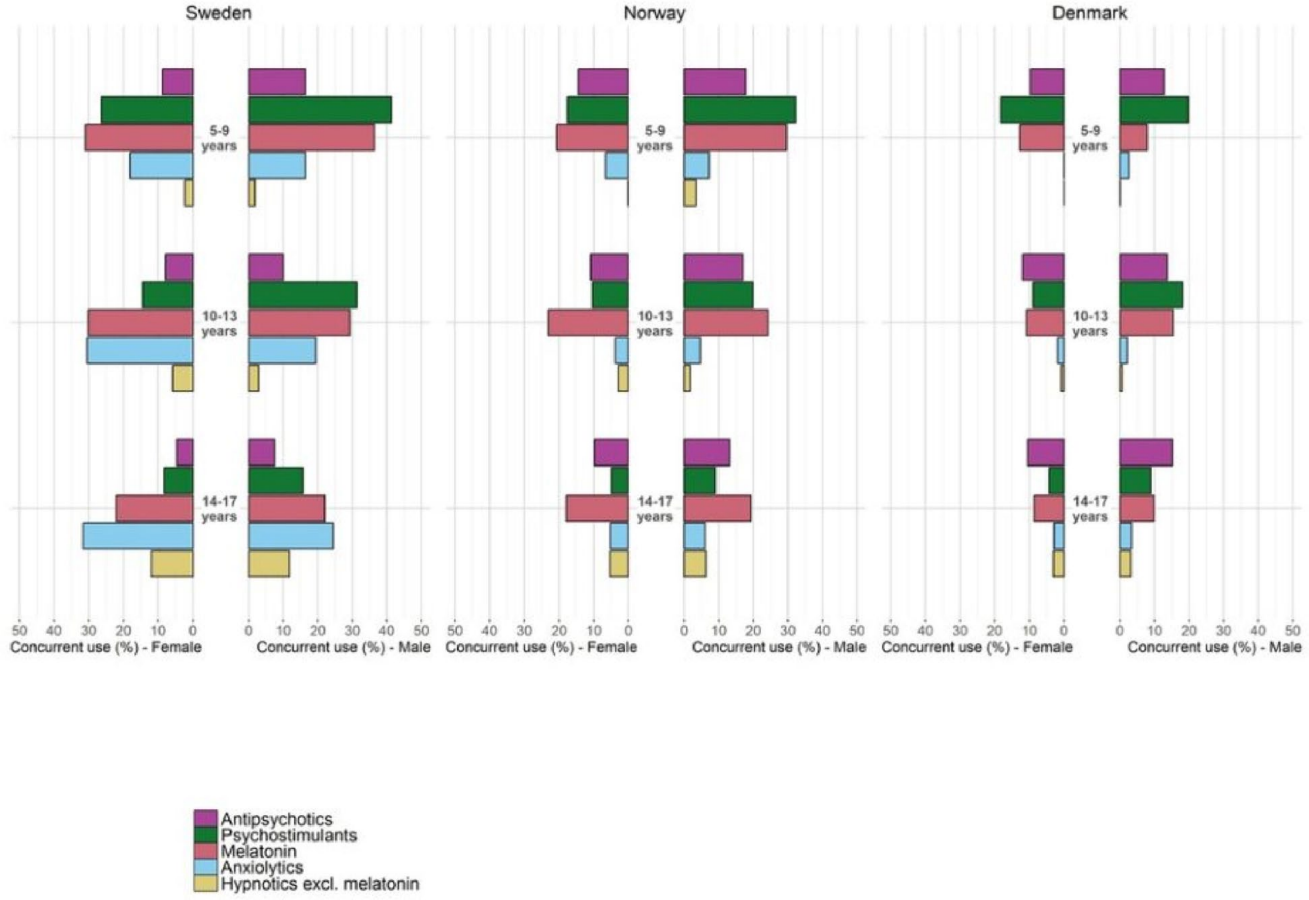
Concomitant use (i.e., filling a prescription 3 months before or 3 months after) (%) of psychotropic drugs among incident users of antidepressants with at least three months follow-up from 2007 to 2018 in Sweden, Norway, and Denmark. Stratified by age and sex

Overall, Swedish antidepressant users were more likely to fill prescriptions for other psychotropics (57%) compared to Norwegian (37%) and Danish (27%) antidepressant users. Concomitant melatonin use was more frequent in Sweden (24%) and Norway (19%) compared to Denmark (9%). The use of anxiolytics was markedly higher in Sweden (28%) compared to Norway (5%) and Denmark (3%). Concomitant psychotropic drug use was more common for boys than girls in Denmark (33% vs 24%) and Norway (41% vs 34%), whereas there was no sex difference between Swedish boys and girls (58% vs 56%). In Fig. 3 we present these figures stratified by sex and age groups.

### Prescribers

Antidepressant treatment was mainly initiated by clinicians working in psychiatry in Sweden (75%) throughout the study period compared to 50% in Denmark. Clinicians working in general practice prescribed 14% of initial prescriptions in Sweden compared to 48% in Denmark (Table 2). In Denmark, there were notable changes over time towards a higher proportion of antidepressant prescriptions being prescribed by clinicians working in psychiatry (in 2013–2018: 69% prescribed by psychiatry and 28% by general practitioners). A markedly larger proportion of prescriptions had missing prescriber information in Denmark (16–17%) compared to Sweden (Table 2).

**Table 2:** Proportion (%, n) of first antidepressant prescriptions prescribed by clinicians working in general practice, psychiatry (adult and child/adolescent), pediatrics, and others

Study period	Clinical setting of the prescriber	Sweden	Denmark
2007–2018*			
	General practice	14.4% (10,141)	47.6% (10,088)
	Psychiatry	74.8% (52,746)	49.7% (10,546)
	Pediatrics	6.4% (4500)	1.1% (233)
	Others	4.4% (3133)	1.6% (338)
2007–2012**			
	General practice	13.8% (3715)	59.1% (7924)
	Psychiatry	73.2% (19,655)	38.5% (5166)
	Pediatrics	6.9% (1852)	1.0% (134)
	Others	6.1% (1636)	1.4% (190)
2013–2018***			
	General practice	14.7% (6426)	27.8% (2164)
	Psychiatry	75.8% (33,091)	69.1% (5380)
	Pediatrics	6.1% (2648)	1.3% (99)
	Others	3.4% (1497)	1.9% (148)

Excluding prescriptions with missing prescriber information in the denominator

*Missing prescriber information: Sweden 0.1% (n = 53); Denmark 15.8% (n = 3991)

**Missing prescriber information: Sweden 0.1% (n = 33); Denmark 15.3% (n = 2426)

***Missing prescriber information: Sweden 0.0% (n = 20); Denmark 16.7% (n = 1565)

## Discussion

We found a marked variation in the number of children and adolescents initiating antidepressant treatment across Scandinavia. Notable was the high and still increasing number of Swedish children and adolescents initiating treatment which was markedly different from the patterns observed in Norway and Denmark. Swedish antidepressant users started treatment at a slightly younger age, were more likely to fill a subsequent antidepressant prescription 12 months after initiation and were also more likely to fill prescriptions of other psychotropics compared to antidepressant users in Denmark and Norway. Antidepressant treatment was primarily initiated by clinicians working in psychiatry in Sweden, whereas this was the case for half of all incident prescriptions in Denmark, although the proportion increased over time. The specific antidepressants prescribed at treatment onset were more in alignment with clinical guideline recommendations and licensed indications in Sweden throughout the study period as opposed to Denmark and Norway.

The steep increase in 10–17-year-old Swedish children and adolescents initiating antidepressant treatment from 2010 onwards was markedly different from the patterns seen among youths from Denmark, where the rates decreased over time, and Norway, where the rates of initiation decreased (14–17-year-old females) or was rather stable over time. The increasing number of Swedish youths initiating antidepressants aligns well to an observed increase in diagnoses of depression and anxiety disorders especially among 10–17-year old girls from 2011 to 2018 [18]. The decreasing number of Norwegian 14–17-year-old adolescent girls initiating antidepressants from 2015 onwards coincides with increased media focus on the increasing use of antidepressants, especially among girls. Likewise, the decreasing number of new antidepressant users in Denmark from 2010 onwards could be influenced by public and clinical skepticism towards antidepressant treatment due to increased media attention on use in children and adolescents [19].

There may be multiple reasons for the observed incidence variations across Scandinavia. First, there could be differences in the prevalence of youths diagnosed with mental disorders where antidepressants are indicated. The Global Burden of Disease study from 2019 [20] shows differences in the prevalence of depression and anxiety among 10–24-year-olds across the three countries. The prevalence of depressive disorders in 10–24-year-olds ranged between 33–34 per 1000 (in Denmark and Norway) and 40 per 1000 (Sweden) while the prevalence of anxiety disorders among 10–24-year-olds ranged between 68 and 71 per 1000 (in Sweden and Denmark) and 93 per 1000 (Norway). Second, prescribing practice differs between the three countries. In Denmark, restrictions from 2007 outline that only child- and adolescent psychiatrists should initiate antidepressant treatment [21, 22], but our data suggest that this has not been strictly adhered to. In Sweden and Norway on the other hand, treatment with antidepressants is recommended to be managed by child- and adolescent psychiatrists, yet there is no legal requirement. Third, differences in the availability of child- and adolescent psychiatrists between the three countries could contribute to variations in youths diagnosed with mental disorders but also in the number of youths offered relevant treatment. Supporting this hypothesis, the number of child- and adolescent psychiatrists per population under 18 years is markedly higher in Sweden compared to Denmark and Norway [23]. In a European comparison of child and adolescent psychiatric training programs the ratio of child- and adolescent psychiatrists to the population under 18 years was 1:5608 in Denmark, 1:4787 in Norway, and 1:2887 in Sweden [23]. The fact that most Swedish children have started antidepressant treatment through contact with a clinician working within psychiatry may also explain the lower age at treatment initiation, as specialists may be more comfortable prescribing antidepressants to children at a younger age than e.g., general practitioners. Finally, differences in clinical practice, e.g., diagnostic and treatment intensity, could contribute to the observed variations. The Swedish clinical guideline recommends use of SSRIs for treatment of moderate depression in children and adolescents to the same extent as cognitive behavioral therapy [24]. This is different from the overall recommendations in Denmark [21] and Norway [25], where antidepressant treatment is recommended for severe conditions, where non-pharmacological interventions are considered insufficient. However, it must be noted that Sweden is the only Scandinavian country with up-to-date disorder specific national clinical guidelines for the treatment of both depression and anxiety including obsessive–compulsive disorder, which makes it difficult to directly compare clinical guidelines and recommendations between countries.

We also observed national differences in use of specific antidepressants, which supports the assumption of differences in clinical practice across countries. There was a high alignment with clinical recommendations and licensed indications of antidepressants in Sweden throughout the study period with a dominant use of sertraline and fluoxetine, whereas this was only found in the latter study years in Denmark and Norway. Furthermore, there was a frequent first-line use of escitalopram in Norway as opposed to Denmark and Sweden, although escitalopram is not approved for use in children and adolescents in the Scandinavian countries. According to recommendations in Norway, however, escitalopram/citalopram may be used in depressed children and adolescents when there is a need for an immediate drug response, e.g., in patients with severe suicidal ideation [25].

Besides differences in utilization of antidepressants across Scandinavia, Swedish antidepressant users were also more likely to fill prescriptions for other psychotropic drugs, although we were not able to distinguish between concomitant use of psychotropics and shifts in treatment in our analysis. This might indicate that Swedish antidepressant users are either suffering from more comorbidity or are more intensively treated for other comorbidities such as ADHD and sleep problems, compared to Danish and Norwegian antidepressant users.

Few epidemiological studies provide data on the duration of antidepressant drug use in children and adolescents [26, 27]. Under the assumption of use of 1 tablet per day, a higher proportion of Swedish youths were covered by an antidepressant prescription at 12 months from initiation compared to Danish and Norwegian youths. The difference between the three countries was however limited for continuous antidepressant treatment.

The major strength of the study is the use of data from nationwide prescription registries with high completeness and validity and with no selection or recall bias [15]. Our study also has limitations. First, we had no information on the indication for treatment in our dataset and antidepressants may be used for non-psychiatric purposes (e.g., TCAs for nocturnal enuresis or migraine). However, limiting our population to individuals with a registered mental disorder would decrease our study population as diagnoses from primary care physicians are not recorded in all the Scandinavian health registries. Second, we had no information on the duration or dose of the single prescription, and we therefore assumed the use of 1 tablet per day and added a grace period of 25% to account for non-compliance and stockpiling. Thus, we may have either overestimated or underestimated the proportion of children in treatment at 6 and 12 months. This problem is, however, most pronounced in the Kaplan–Meier survival analysis while the PPC is less sensitive to these assumptions [17]. Third, as our data were based on filled prescriptions, we do not know to which extent the antidepressants were consumed, and it is likely that the true prevalence and incidence of use is lower. Fourth, in our analysis of concomitant psychotropic drug use, we only captured prescription drugs dispensed at pharmacies and drugs obtained through other channels was not registered. However, all drugs included in the analysis were available by prescription only during the study period. Fifth, there was a large proportion of prescriptions with missing prescriber information in Denmark (16–17%). Although the prescriber information is considered valid in the Danish data sources [28], we cannot rule out that some prescriptions with missing information was prescribed by clinicians working in psychiatry which may attenuate some of the differences observed between Denmark and Sweden. Finally, our study period ended in 2018, and the utilization patterns of antidepressants has changed since then. The COVID-19 pandemic and the national lockdown was associated with an increased use of antidepressants among Danish youths [29]. This was also observed among Norwegian youths in late 2020 [30]. Contrary to most countries, Sweden did not impose lockdowns and they experienced a rise in antidepressant use among youths in March 2020, probably reflecting stockpiling [30].

## Conclusion

We examined antidepressant use among Scandinavian children and adolescents during 2007–2018 and found remarkable national differences with a high and increasing number of Swedish youths initiating antidepressant treatment, which differed from the patterns observed in Norway and Denmark. Swedish antidepressant users were more likely to use other psychotropics and to refill antidepressant prescriptions after one year. This could suggest lower thresholds for antidepressant treatment in Sweden compared to Denmark and Norway, which is partly supported by the Swedish clinical guideline that recommends SSRIs to the same level as cognitive behavioral therapy for moderate depression in childhood. However, we found that antidepressants were primarily initiated by physicians working within psychiatry in Sweden throughout the study period. The choice of first antidepressant drug was more in line with clinical guidelines and licensing status of antidepressants in Sweden compared to Denmark and Norway throughout the study period. Sweden has a higher availability of child and adolescent psychiatric specialists as well as updated disorder specific treatment guidelines for depression and anxiety disorders as opposed to Denmark and Norway. Altogether, the high and increasing use of antidepressants in Sweden could therefore also reflect better access to qualified psychiatric health care compared to Denmark and Norway. Further studies are needed to assess this hypothesis.

## Supplementary Information

Below is the link to the electronic supplementary material.

Supplementary file1 (DOCX 77 KB)

## Data Availability

Due to Nordic legislation, individual-level data are not available. Deidentified data can be made available for authorized researchers after application to Forskerservice at the Danish Health Data Authority (in Denmark), Helsedataservice at the Norwegian Institute of Public Health (in Norway), and the National Board of Health and Welfare (in Sweden). PBJ had full access to Danish data and aggregated data from Norway and Sweden. RS had full access to Norwegian data. JR had full access to Swedish data.

## Code availability

Coding scripts are available upon request.

## References

[1] Dubrall D, Fekete S, Leitzen S et al (2023) Selective serotonin reuptake inhibitors and suicidality in children and young adults: analyses of pharmacovigilance databases. BMC Pharmacol Toxicol 24:22. 10.1186/s40360-023-00664-z 37004083 10.1186/s40360-023-00664-z PMC10067298

[2] European Medicines Agency (2005) European Medicines Agency finalises review of antidepressants in children and adolescents

[3] Hammad TA, Laughren T, Racoosin J (2006) Suicidality in pediatric patients treated with antidepressant drugs. Arch Gen Psychiatry 63:332–339. 10.1001/archpsyc.63.3.332 16520440 10.1001/archpsyc.63.3.332

[4] Sharma T, Guski LS, Freund N, Gøtzsche PC (2016) Suicidality and aggression during antidepressant treatment: systematic review and meta-analyses based on clinical study reports. BMJ 352:i65. 10.1136/bmj.i65 26819231 10.1136/bmj.i65 PMC4729837

[5] Vitiello B, Swedo S (2004) Antidepressant medications in children. N Engl J Med 350:1489–1491. 10.1056/NEJMp038248 15071123 10.1056/NEJMp038248

[6] Piovani D, Clavenna A, Bonati M (2019) Prescription prevalence of psychotropic drugs in children and adolescents: an analysis of international data. Eur J Clin Pharmacol. 10.1007/s00228-019-02711-3 31270564 10.1007/s00228-019-02711-3

[7] Bachmann CJ, Aagaard L, Burcu M et al (2016) Trends and patterns of antidepressant use in children and adolescents from five western countries, 2005–2012. Eur Neuropsychopharmacol J Eur Coll Neuropsychopharmacol 26:411–419. 10.1016/j.euroneuro.2016.02.001 10.1016/j.euroneuro.2016.02.001 26970020

[8] Hartz I, Skurtveit S, Hjellvik V et al (2016) Antidepressant drug use among adolescents during 2004–2013: a population-based register linkage study. Acta Psychiatr Scand 134:420–429. 10.1111/acps.12633 27571234 10.1111/acps.12633 PMC5096062

[9] Wesselhoeft R, Jensen PB, Talati A et al (2019) Trends in antidepressant use among children and adolescents: a Scandinavian drug utilization study. Acta Psychiatr Scand. 10.1111/acps.13116 31618447 10.1111/acps.13116

[10] Holm S, Liss PE, Norheim OF (1999) Access to health care in the Scandinavian countries: ethical aspects. Health Care Anal HCA J Health Philos Policy 7:321–330. 10.1023/A:1009460010196 10.1023/A:1009460010196 10787795

[11] European Medicines Agency. https://www.ema.europa.eu/en

[12] Wettermark B, Hammar N, Fored CM et al (2007) The new Swedish Prescribed Drug Register–opportunities for pharmacoepidemiological research and experience from the first six months. Pharmacoepidemiol Drug Saf 16:726–735. 10.1002/pds.1294 16897791 10.1002/pds.1294

[13] Furu K (2008) Establishment of the nationwide Norwegian Prescription Database (NorPD)—new opportunities for research in pharmacoepidemiology in Norway. Nor Epidemiol. 10.5324/nje.v18i2.23

[14] Pottegård A, Schmidt SAJ, Wallach-Kildemoes H et al (2017) Data resource profile: the Danish National Prescription Registry. Int J Epidemiol 46:798–798f. 10.1093/ije/dyw213 27789670 10.1093/ije/dyw213 PMC5837522

[15] Furu K, Wettermark B, Andersen M et al (2010) The Nordic countries as a cohort for pharmacoepidemiological research. Basic Clin Pharmacol Toxicol 106:86–94. 10.1111/j.1742-7843.2009.00494.x 19961477 10.1111/j.1742-7843.2009.00494.x

[16] WHOCC—ATC/DDD Index. https://www.whocc.no/atc_ddd_index/. Accessed 6 July 2023

[17] Rasmussen L, Pratt N, Hansen MR et al (2018) Using the “proportion of patients covered” and the Kaplan-Meier survival analysis to describe treatment persistence. Pharmacoepidemiol Drug Saf 27:867–871. 10.1002/pds.4582 29952045 10.1002/pds.4582

[18] Swedish National Board of Health and Welfare (2018) Inrapporterade depressioner och ångestsyndrom bland barn och unga vuxna – utvecklingen till och med 2018

[19] Green Lauridsen M, Kälvemark Sporrong S (2018) How does media coverage effect the consumption of antidepressants? A study of the media coverage of antidepressants in Danish online newspapers 2010–2011. Res Soc Adm Pharm 14:638–644. 10.1016/j.sapharm.2017.07.011 10.1016/j.sapharm.2017.07.011 28811152

[20] Institute for Health Metrics and Evaluation (IHME). GBD compare data visualization. Seattle, WA: IHME, University of Washington, 2020. http://vizhub.healthdata.org/gbd-compare. Accessed 12 Jan 2024

[21] The Ministry of the Interior and Health (2019) Vejledning om medikamentel behandling af børn og unge med psykiske lidelser [Guideline on the use of medicine in children and adolescents with psychiatric disorders]

[22] The Ministry of the Interior and Health (2007) Vejledning om medikamentel behandling af børn og unge med psykiske lidelser [Guideline on the use of medicine in children and adolescents with psychiatric disorders]. https://www.retsinformation.dk/eli/retsinfo/2007/10332. Accessed 24 Feb 2015

[23] Barrett E, Jacobs B, Klasen H et al (2020) The child and adolescent psychiatry: study of training in Europe (CAP-STATE). Eur Child Adolesc Psychiatry 29:11–27. 10.1007/s00787-019-01416-3 31845068 10.1007/s00787-019-01416-3

[24] Swedish National Board of Health and Welfare (2021) Nationella riktlinjer för vård vid depression och ångestsyndrom, 2021. [National guidelines for care in cases of depression and anxiety disorders]

[25] Norsk barne- og ungdomspsykiatrisk forening Faglig veileder for barne- og ungdomspsykiatri [Guidance in child- and adolescent psychiatry], 4. utgave

[26] Saastamoinen LK, Wallin M, Lavikainen P et al (2012) Treatment duration with selective serotonin reuptake inhibitors among children and adolescents in Finland: a nationwide register study. Eur J Clin Pharmacol 68:1109–1117. 10.1007/s00228-012-1233-6 22328104 10.1007/s00228-012-1233-6

[27] Pottegård A, Zoëga H, Hallas J, Damkier P (2014) Use of SSRIs among Danish children: a nationwide study. Eur Child Adolesc Psychiatry 23:1211–1218. 10.1007/s00787-014-0523-1 24493268 10.1007/s00787-014-0523-1

[28] Rasmussen L, Valentin J, Gesser KM et al (2016) Validity of the prescriber information in the Danish National Prescription Registry. Basic Clin Pharmacol Toxicol. 10.1111/bcpt.12610 27098169 10.1111/bcpt.12610

[29] Bliddal M, Rasmussen L, Andersen JH et al (2023) Psychotropic medication use and psychiatric disorders during the COVID-19 pandemic among Danish children, adolescents, and young adults. JAMA Psychiat 80:176–180. 10.1001/jamapsychiatry.2022.4165 10.1001/jamapsychiatry.2022.4165 PMC9856810 36515919

[30] Tiger M, Wesselhoeft R, Karlsson P et al (2023) Utilization of antidepressants, anxiolytics, and hypnotics during the COVID-19 pandemic in Scandinavia. J Affect Disord 323:292–298. 10.1016/j.jad.2022.11.068 36442654 10.1016/j.jad.2022.11.068 PMC9691511

