# Supplementary material for: Treatment patterns of antidepressants in children and adolescents in Scandinavia
Source: Eur Child Adolesc Psychiatry. 2024 Apr 29;34(1):159–67. doi: 10.1007/s00787-024-02433-7 (PMC11805761; doi:10.1007/s00787-024-02433-7)
Supplement: Supplementary file 1 — Supplementary file1 (DOCX 77 KB) [file 787_2024_2433_MOESM1_ESM.docx]

**Treatment patterns of antidepressants in children and adolescents in Scandinavia**Running title: Use of antidepressants in Scandinavian youths

**European Child and Adolescent Psychiatry**

Authors:

Lotte Rasmussen^1^ ORCID: 0000-0001-5962-6647
Peter Bjødstrup Jensen^1^
 Johan Reutfors^3^ ORCID: 0000-0003-1372-4262
 Kari Furu^4^ ORCID: 0000-0003-2245-0179
Svetlana Skurtveit^4^ ORCID: 0000-0001-7525-9701
Randi Selmer^4^ ORCID: 0000-0003-2245-0179
Per Damkier^6,7^ ORCID: 0000-0003-0591-7187
Mette Bliddal^1,8^ ORCID: 0000-0002-7637-3730
Rikke Wesselhoeft^1,2^ ORCID: 0000-0001-9700-2739

1) Clinical Pharmacology, Pharmacy and Environmental Medicine, Department of Public Health, University of Southern Denmark.

2) Research Unit of Child and Adolescent Mental Health, Institute for Clinical Research, University of Southern Denmark

3) Centre for Pharmacoepidemiology, Clinical Epidemiology Division, Department of Medicine Solna, Karolinska Institutet, Stockholm, Sweden

4) Department of Chronic Diseases, Norwegian Institute of Public Health, Norway

6) Department of Clinical Pharmacology, Odense University Hospital DK-5000 Odense C, Denmark

7) Department of Clinical Research, University of Southern Denmark, DK-5000 Odense, Denmark

8) OPEN Research Unit, Department of Clinical Research, University of Southern Denmark, DK-5000 Odense C, Denmark

**Supporting Information**

**Fig. S1**

Incidence rate (new users per 1,000 person-years) of antidepressant use according to type of antidepressant from 2007-2018 stratified by country. A: all antidepressants, B: selective serotonin reuptake inhibitors (SSRIs), C: tricyclic antidepressants (TCAs), D: other antidepressants.


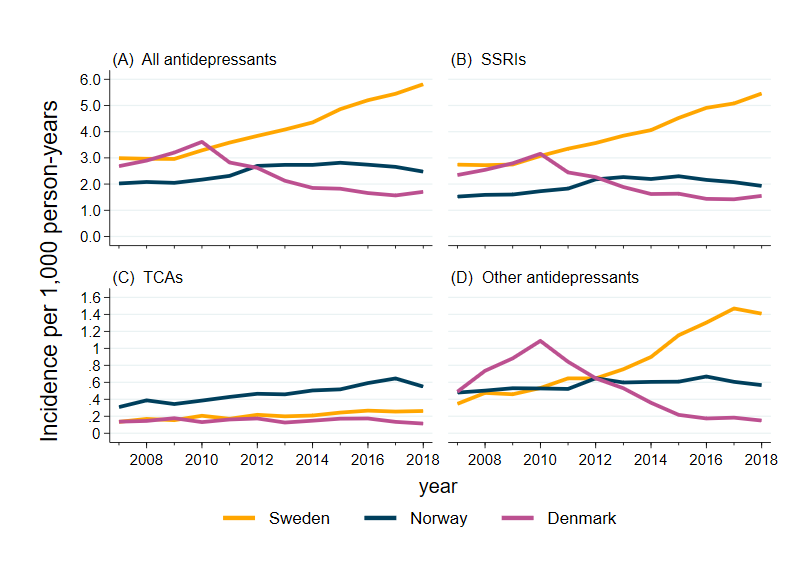


**Table S1a**

Incidence rate (new users per 1,000 person-years) of antidepressant use in Sweden from 2007-2018 overall, according to age, and female to male ratio (F/M).

|  | **2007** | **2008** | **2009** | **2010** | **2011** | **2012** | **2013** | **2014** | **2015** | **2016** | **2017** | **2018** |
| --- | --- | --- | --- | --- | --- | --- | --- | --- | --- | --- | --- | --- |
| **Overall IR** | 2.99 | 2.97 | 2.96 | 3.29 | 3.58 | 3.84 | 4.08 | 4.35 | 4.86 | 5.20 | 5.45 | 5.81 |
| **F/M ratio** | 1.85 | 1.75 | 1.71 | 1.62 | 1.60 | 1.57 | 1.67 | 1.79 | 1.85 | 2.01 | 2.00 | 1.79 |
| **Age 5-9** | 0.23 | 0.24 | 0.24 | 0.34 | 0.38 | 0.47 | 0.43 | 0.48 | 0.54 | 0.55 | 0.63 | 0.63 |
| **F/M ratio** | 0.58 | 0.62 | 0.32 | 0.52 | 0.57 | 0.45 | 0.42 | 0.54 | 0.43 | 0.44 | 0.51 | 0.55 |
| **Age 10-13** | 1.71 | 1.88 | 1.69 | 2.14 | 2.38 | 2.61 | 2.82 | 3.30 | 3.65 | 4.17 | 4.33 | 4.74 |
| **F/M ratio** | 0.92 | 0.91 | 0.90 | 0.92 | 0.98 | 0.91 | 0.94 | 1.03 | 1.17 | 1.22 | 1.33 | 1.19 |
| **Age 14-17** | 6.61 | 6.49 | 6.84 | 7.59 | 8.60 | 9.45 | 10.42 | 10.93 | 12.35 | 12.89 | 13.33 | 14.05 |
| **F/M ratio** | 2.27 | 2.15 | 2.08 | 1.99 | 1.94 | 2.00 | 2.16 | 2.37 | 2.40 | 2.73 | 2.60 | 2.29 |

**Table S1b**

Incidence rate (new users per 1,000 person-years) of antidepressant use in Norway from 2007-2018 overall, according to age, and female to male ratio (F/M).

|  | **2007** | **2008** | **2009** | **2010** | **2011** | **2012** | **2013** | **2014** | **2015** | **2016** | **2017** | **2018** |
| --- | --- | --- | --- | --- | --- | --- | --- | --- | --- | --- | --- | --- |
| **Overall IR** | 2.02 | 2.08 | 2.05 | 2.17 | 2.31 | 2.69 | 2.73 | 2.73 | 2.81 | 2.74 | 2.66 | 2.48 |
| **F/M ratio** | 1.80 | 1.89 | 1.72 | 1.70 | 2.01 | 2.09 | 2.11 | 2.30 | 2.36 | 2.35 | 2.27 | 2.03 |
| **Age 5-9** | 0.18 | 0.18 | 0.15 | 0.21 | 0.18 | 0.14 | 0.17 | 0.14 | 0.15 | 0.12 | 0.14 | 0.13 |
| **F/M ratio** | 0.39 | 0.36 | 0.52 | 0.51 | 0.58 | 0.56 | 0.40 | 0.83 | 0.40 | 0.59 | 0.62 | 0.76 |
| **Age 10-13** | 0.75 | 0.80 | 0.93 | 0.89 | 0.88 | 1.03 | 0.88 | 0.92 | 0.95 | 0.89 | 0.89 | 0.87 |
| **F/M ratio** | 0.57 | 0.61 | 0.77 | 1.01 | 0.63 | 0.73 | 0.98 | 0.93 | 0.82 | 0.93 | 1.19 | 1.12 |
| **Age 14-17** | 5.48 | 5.58 | 5.38 | 5.72 | 6.17 | 7.25 | 7.54 | 7.57 | 7.87 | 7.82 | 7.59 | 7.09 |
| **F/M ratio** | 2.28 | 2.43 | 2.10 | 1.97 | 2.55 | 2.58 | 2.45 | 2.68 | 2.86 | 2.74 | 2.57 | 2.26 |

**Table S1c**

Incidence rate (new users per 1,000 person-years) of antidepressant use in Denmark from 2007-2018 overall, according to age, and female to male ratio (F/M).

|  | **2007** | **2008** | **2009** | **2010** | **2011** | **2012** | **2013** | **2014** | **2015** | **2016** | **2017** | **2018** |
| --- | --- | --- | --- | --- | --- | --- | --- | --- | --- | --- | --- | --- |
| **Overall IR** | 2.68 | 2.90 | 3.20 | 3.61 | 2.82 | 2.62 | 2.13 | 1.85 | 1.82 | 1.66 | 1.57 | 1.70 |
| **F/M ratio** | 1.99 | 2.17 | 1.98 | 2.24 | 1.87 | 2.13 | 2.24 | 2.10 | 2.06 | 2.14 | 2.11 | 1.92 |
| **Age 5-9** | 0.22 | 0.23 | 0.26 | 0.28 | 0.22 | 0.16 | 0.12 | 0.11 | 0.10 | 0.10 | 0.07 | 0.07 |
| **F/M ratio** | 0.37 | 0.39 | 0.87 | 0.56 | 0.82 | 0.39 | 0.94 | 0.35 | 0.65 | 0.55 | 0.60 | 0.46 |
| **Age 10-13** | 1.14 | 1.17 | 1.30 | 1.42 | 1.35 | 1.23 | 1.05 | 1.02 | 0.93 | 0.83 | 0.88 | 0.91 |
| **F/M ratio** | 0.92 | 0.86 | 0.97 | 1.05 | 0.89 | 0.93 | 0.83 | 0.80 | 0.81 | 0.93 | 1.21 | 1.16 |
| **Age 14-17** | 7.37 | 7.88 | 8.53 | 9.55 | 7.22 | 6.76 | 5.52 | 4.74 | 4.74 | 4.35 | 4.05 | 4.43 |
| **F/M ratio** | 2.46 | 2.73 | 2.30 | 2.69 | 2.24 | 2.65 | 2.85 | 2.80 | 2.59 | 2.64 | 2.47 | 2.21 |

**Table S2**

Proportion (%, n) of incident users of antidepressants in continuous treatment (estimated by Kaplan-Meier (KM) survival analysis) and covered by a prescription (estimated by the PPC method) after 6 and 12 months. Stratified by age and country.

|  | Sweden | | Norway | | Denmark | |
| --- | --- | --- | --- | --- | --- | --- |
|  | PPC | KM | PPC | KM | PPC | KM |
| At 6 months |  |  |  |  |  |  |
| Total | 72.7 % (43749) | 55.0 % (33163) | 58.4 % (12664) | 48.9 % (10645) | 65.6 % (15072) | 50.8 % (11693) |
| Age 5-9 | 62.8 % (1317) | 44.8 % (942) | 55.4 % (293) | 47.5 % (251) | 52.9 % (315) | 36.1 % (215) |
| Age 10-13 | 73.6 % (9127) | 57.7 % (7167) | 62.9 % (1517) | 55.1 % (1334) | 72.4 % (2356) | 56.4 % (1836) |
| Age 14-17 | 72.0 % (32875) | 54.7 % (25054) | 57.6 % (10809) | 48.1 % (9060) | 64.3 % (12290) | 50.3 % (9642) |
|  |  |  |  |  |  |  |
| At 12 months |  |  |  |  |  |  |
| Total | 57.7 % (34630) | 34.4 % (20708) | 39.5 % (8565) | 25.6 % (5556) | 49.0 % (11227) | 30.9 % (7110) |
| Age 5-9 | 47.7 % (999) | 29.2 % (613) | 38.5 % (203) | 29.7 % (158) | 38.6 % (229) | 23.2 % (139) |
| Age 10-13 | 58.4 % (7228) | 39.6 % (4907) | 45.6 % (1097) | 33.8 % (815) | 57.0 % (1850) | 37.1 % (1206) |
| Age 14-17 | 55.3 % (25204) | 33.2 % (15188) | 37.9 % (7105) | 24.4 % (4583) | 46.4 % (8864) | 30.1 % (5765) |
